# A Moderated Mediation Model Linking Speech-in-Noise Exposure, Speech Perception in Noise Ability, Loneliness, and Depression

**DOI:** 10.1044/2026_JSLHR-25-00615

**Published:** 2026-06-24

**Authors:** Erik Jorgensen, Megan Werner, Lucas Modahl, Sara Misurelli

**Affiliations:** a Department of Communication Sciences and Disorders, University of Wisconsin–Madison; b Department of Otolaryngology-Head and Neck Surgery, School of Medicine and Public Health, University of Wisconsin–Madison

## Abstract

**Purpose:**

Hearing loss is associated with elevated levels of loneliness and depression, but the factors accounting for these associations remain unclear. This study examined whether real-world hearing behaviors—time spent in speech-in-noise (SiN) environments and speech perception in noise (SPIN) ability—are associated with loneliness and depression in young adults with normal hearing, using a moderated mediation model.

**Method:**

Fifty-four participants with audiometrically normal hearing completed standardized assessments of SPIN ability (Quick Speech-in-Noise test), loneliness (UCLA Loneliness Scale), and depression (Patient Health Questionnaire-9). Participants wore audio recorders for 1 week, and an open-source deep neural network (Yet Another Mobile Network) classified recordings to estimate SiN exposure time. Regression-based moderated mediation analyses tested whether the association between SiN exposure and depression was mediated by loneliness and moderated by SPIN ability.

**Results:**

Greater time in SiN was associated with lower loneliness, particularly among individuals with average or better SPIN ability. In contrast, participants with poorer SPIN ability showed weaker associations, with high exposures sometimes associated with greater loneliness. Loneliness was positively associated with depression scores. A significant moderated mediation association emerged: The indirect association between SiN exposure and depression via loneliness was statistically significant only for individuals with approximately average or better SPIN ability.

**Conclusions:**

These findings are consistent with a person–environment fit perspective: Individuals whose hearing abilities align well with the demands of their environments tend to show better social–emotional health outcomes, whereas those with a mismatch between SPIN ability and SiN exposure show weaker or even reversed associations. This study illustrates how hearing-related behaviors and hearing abilities may be associated with social–emotional well-being and underscores the importance of considering both individual ability and everyday listening environments when designing and evaluating hearing health interventions.

Hearing loss is associated with increased levels of negative social–emotional health outcomes—particularly loneliness and depression (Chakrabarty et al., 2024; Döge et al., 2025; G. R. Lee & Ishii-Kuntz, 1987; Jayakody et al., 2022; Wagner et al., 2025; for reviews, see Lawrence et al., 2020; Li et al., 2025; Shukla et al., 2020; Wei et al., 2024). However, why hearing loss is associated with loneliness and depression is not well understood. Loneliness is the subjective, unpleasant feeling that arises when someone perceives that their “social needs are not being met by the quantity or especially the quality of one's social relationships” (Hawkley & Cacioppo, 2010; Russell et al., 1978). Persistent loneliness can lead to or increase levels of depression, a prolonged experience of low mood, including feeling sad or empty, and a loss of pleasure or interest in doing things (Achterbergh et al., 2020; Kroenke et al., 2001; S. L. Lee et al., 2021; Mann et al., 2022; Otte et al., 2016). If the associations between hearing loss and social–emotional health are related to hearing loss itself and not some shared underlying factor (e.g., Keesom & Hurley, 2020), then the connection should be evident through hearing-related behaviors in daily life. Many social interactions take place in acoustically challenging speech-in-noise (SiN) environments, where individuals with hearing loss experience more effortful and poorer quality communication (Beechey et al., 2019; Ellis et al., 2024; Heffernan et al., 2016; Miles et al., 2023; Petersen et al., 2022; Pichora-Fuller et al., 2016, 2017). These difficulties can prompt disengagement during or avoidance of noisy social situations (Barker et al., 2017; Heffernan et al., 2016; Herrmann & Johnsrude, 2020; Motala et al., 2024; Palmer et al., 2016; Resnick et al., 1997), creating a perceived mismatch between desired and realized social connections that may contribute to loneliness and, potentially, depression (Courtin & Knapp, 2017; Jiang et al., 2022; S. L. Lee et al., 2021; Mann et al., 2022; Shukla et al., 2020; Zhang et al., 2018). Although this theoretical pathway is widely discussed, little empirical evidence directly supports it, in part due to the lack of controlled trials investigating the consequences of hearing loss (Munro et al., 2021; Tang et al., 2025) and the challenge of measuring hearing-related behaviors in daily life (Keidser et al., 2020; Neal et al., 2022). The purpose of this study was to begin building a framework around and provide some evidence for this theory by characterizing associations between time spent in SiN environments, speech perception in noise (SPIN) ability, loneliness, and depression in an observational study of hearing-related behavior in young adults with audiometrically normal hearing.

Understanding how hearing-related behaviors may shape social–emotional outcomes requires characterizing those behaviors, ideally in daily life. This poses some methodological challenges. Most studies linking hearing to loneliness and depression have relied on self-report hearing status (Shukla et al., 2020). A recent study (Döge et al., 2025) used objective audiometric thresholds to quantify the link between hearing loss, loneliness (using a single-item question), and depression (quantified by the Patient Health Questionnaire-9 [PHQ-9]) in a large cohort of adults. They found significant increases in odds of loneliness for individuals with severe to profound hearing loss and significant increases in odds of depression for individuals with mild to severe hearing loss. Although more reliable than self-report hearing status, audiometry may not sufficiently capture real-world hearing ability, particularly in the noisy environments where communication challenges are most likely to impact social well-being. Another recent study investigated the relationship between hearing loss and loneliness using both audiometry data and SPIN ability (Huang et al., 2024). They showed that greater hearing loss (quantified by pure-tone average) was associated with more loneliness (quantified by the UCLA Loneliness Scale [UCLA-LS]). They also showed that better SPIN ability (quantified by the Quick Speech-in-Noise [QuickSIN] test) was associated with larger and more diverse social networks. However, they did not find an association between SPIN ability and loneliness, suggesting that the relationship between SPIN ability and loneliness may be more complex than a direct correlative association.

An important piece missing from prior work is an estimation of SiN exposure. Measuring how much time individuals spend in different acoustic environments in daily life is difficult. The simplest method for estimating time spent in different environments is to use retrospective questionnaires, such as the Auditory Lifestyle and Demand Questionnaire (Gatehouse et al., 1999, 2003; Jorgensen et al., 2023) or the Hearing-Related Lifestyle Questionnaire (Lelic et al., 2022). However, these measures have important limitations: They lack granularity and are prone to recall and reporting biases (Lelic et al., 2022; Shiffman et al., 2008; Wu et al., 2020). Another option is to use ecological momentary assessment (EMA), where individuals report on their listening activities or environments frequently throughout the day (Wu et al., 2018). EMA offers more granularity and validity than retrospective questionnaires, but it systematically underestimates time in SiN for the simple reason that most individuals do not complete surveys when they are in social situations (Schinkel-Bielefeld et al., 2020). A better option is to use wearable sensors that measure the acoustics of the environment in real time, such as dosimeters, recorders, or hearing aids (Benítez-Barrera et al., 2020; Humes et al., 2018; Klein et al., 2018; Jorgensen, Tufts, & Skoe, 2025; Jorgensen et al., 2023; Wu & Bentler, 2012). Dosimeters and hearing aids can provide simple sound pressure levels, which are useful indicators of time spent in noisy environments. However, sound levels alone are limited because they cannot differentiate SiN from other sounds with high sound levels (Jorgensen, Tufts, & Skoe, 2025; Wu & Bentler, 2012). Recording devices like the Language Environment Analysis (LENA) system or hearing aids with sound classification are able to provide estimates of time spent in SiN. Studies using such devices have found that older adults spend between 5% and 10% of their time in noisy environments (Humes et al., 2018; Klein et al., 2018). These technologies have many advantages, as they are wearable and include built-in acoustic environment classification. However, they are relatively expensive, the algorithms they use to make the sound classifications are proprietary, and the accuracy of their classification is variable (Cristia et al., 2021; Meera et al., 2025; Yellamsetty et al., 2021). Advancements in sound classification using machine learning techniques have opened new possibilities for flexible, granular characterization of acoustic environments with open-source algorithms and no specialized equipment (Bansal & Garg, 2022; Piczak, 2015; Tsalera et al., 2021). The present study leverages these techniques to estimate SiN exposure in daily life and relate it to SPIN ability and social–emotional well-being.

Understanding the links between hearing and social–emotional health outcomes has important implications. Hearing loss, loneliness, and depression are major health epidemics among older adults (Haile et al., 2024; Jalali et al., 2024). Beyond the intrinsically negative impacts of loneliness and depression on daily life, loneliness following hearing loss has been suggested as one potential mechanism linking hearing loss and dementia (Holwerda et al., 2014; Penninkilampi et al., 2018; Sommerlad et al., 2023). Improvements in social–emotional health are therefore critical goals of auditory rehabilitation (Chisolm et al., 2007; McRackan et al., 2018; Timmer et al., 2023). Cochlear implants have been shown to lead to improvements in quality of life and reductions in loneliness and depression (Choi et al., 2016; Contrera et al., 2017; McRackan et al., 2018, 2023; Mo et al., 2005). Improvements in social–emotional health following cochlear implantation are not predicted by improvements in speech perception, and impacts on individual social–emotional health outcomes may be a more sensitive and ecological measure of cochlear implant success than traditional speech tests (Brown et al., 2025; McRackan et al., 2018, 2023; Moberly et al., 2018). Hearing aids improve quality of life (Avierinos et al., 2024; Chisolm et al., 2007), but the limited research on whether hearing aids improve loneliness and depression is equivocal (Döge et al., 2025; Reed et al., 2025; Singh et al., 2025; for a review, see Bott & Saunders, 2021). Understanding how hearing loss may be associated with loneliness and depression, and why cochlear implants—but not hearing aids—appear to provide clear social–emotional benefit, requires quantifying the role of hearing-related behaviors in daily life and, eventually, the role of hearing health interventions on those behaviors.

We propose that hearing-related behaviors—specifically time spent in SiN environments and the ability to participate in these settings—could underlie the link between hearing and risk of loneliness and depression through moderated mediation. Specifically, we hypothesize that spending less time in SiN environments is associated with increased loneliness, and that this relationship is moderated by SPIN ability: Individuals with better SPIN ability may experience more social–emotional benefit with time spent in SiN contexts, whereas those with poorer SPIN ability may not. In turn, we expect greater loneliness to be associated with greater depression, such that SiN exposure and SPIN ability may be indirectly associated with depression via loneliness. We aimed to begin assessing this model by focusing on individual differences in SPIN ability in young adults with normal hearing. We focused on young adults for this initial study because this group provides a clearer test of the proposed associations without the influence of age or age-related hearing loss. Young adults also show meaningful variability in SPIN ability despite having clinically normal hearing, making it possible to examine how differences in hearing ability relate to everyday hearing behaviors and social–emotional outcomes. Studying this population first allowed us to evaluate the structure of the model under well-controlled conditions before extending it to older adults and individuals with hearing loss. We used open-source machine learning tools to quantify SiN exposure from longform audio recordings of daily life, administered the QuickSIN to assess SPIN ability, and measured loneliness and depression using validated self-report questionnaires (UCLA-LS and PHQ-9). We then used a multiple regression approach to examine the moderated mediation pattern of associations among SiN exposure, SPIN ability, loneliness, and depression.

## Method

### General Procedure

This study was part of a multidimensional investigation of daily life and its relationship to hearing. The design was cross-sectional. Participants attended two lab visits separated by 1 week. During the initial visit, participants completed intake, otoscopy, pure-tone audiometry, and SPIN assessment. Then, they were trained on the use of the recorder and EMA. Throughout the intervening week, participants wore the recorder for as many waking hours as possible and completed EMAs periodically throughout the day. At the second visit, participants returned the equipment, completed the retrospective assessments, and were paid for their participation. All participants began and ended the study on a weekday, so that data collection spanned both weekdays and a weekend. One week of data collection is likely sufficient to represent the auditory life of a college-aged listener, but data collection on all weekdays is required as auditory lifestyle can vary between weekdays and weekends (Jorgensen, Tufts, & Skoe, 2025).

### Participants

Data from 54 participants were analyzed. Initially, 55 participants were recruited, but one withdrew over privacy concerns with the audio recorder. Participants included young adults between 18 and 26 years (*M* = 21.31, *SD* = 2.36). All participants had normal hearing (air-conduction thresholds ≤ 20 dB HL from 0.25 to 8 kHz bilaterally). Participants identified as 38 females, 13 males, and 3 nonbinary. All participants were fluent in English. Recruitment occurred through mass e-mail, and participants were scheduled on a first-come, first-served basis. The University of Wisconsin–Madison institutional review board approved this study. All participants provided written consent to participate in the study. Data were collected between July of 2023 and April of 2024.

### SPIN Assessment

SPIN ability was measured using the QuickSIN (Killion et al., 2004). The test was administered in the sound field from a speaker (KEF Q350) at 0° azimuth in a soundproof booth. The presentation level was 70 dBA. During the QuickSIN, participants repeat back six target sentences per list, where the signal-to-noise ratio (SNR) becomes smaller by 5 dB each list from 25 to 0 dB SNR. Participants are scored on key words correct, and a dB SNR loss is calculated. The dB SNR loss quantifies the difference between the participant's SNR50, the SNR at which they perceive approximately 50% of the key words correctly, and a normative sample. Lower SNR loss scores indicate better SPIN ability, with negative values indicating better-than-average SPIN ability. Participants wore a talkback microphone on their collar so their responses were audible and clear. Participants were instructed that they would hear a sentence from a woman with several talkers in the background. They were instructed to try and repeat as much of the sentence as they could. Participants first completed a practice list. Then, participants completed two test lists, drawn randomly from the set of 12 equivalent lists. Participants were scored on key words correct, and dB SNR loss scores were calculated by subtracting key words correct from 25.5. The average of the two test lists was taken as the participants' dB SNR loss. List randomization, presentation, scoring, and automatic dB SNR loss calculation were performed using custom MATLAB scripts.

### Social–Emotional Health Assessment

Depression and loneliness were assessed using standardized self-report questionnaires. Questionnaires were administered through Qualtrics on a computer in the laboratory during Visit 2 of the study. Depression was measured using the PHQ-9 (Kroenke et al., 2001). The PHQ-9 is a widely used screening questionnaire for depression. It has been used in many clinical and research applications, including to investigate the relationship between depression and hearing loss (see Lawrence et al., 2020, for a review). The PHQ-9 asks participants to report how bothered they have been by various problems over the prior 2 weeks—for example, how much they have been bothered by feeling down, depressed, or hopeless. Participants respond by indicating they were not at all bothered, bothered on several days, bothered on more than half the days, or bothered nearly every day. Responses are scored as 0, 1, 2, or 3, respectively. The scores are summed and treated continuously. Higher scores indicate greater depression severity.

Loneliness was measured using the UCLA-LS (Russell et al., 1978, 1980). The UCLA-LS is the most widely used measure of loneliness (Yanguas et al., 2018). It has been used extensively to measure the effects of many phenomena on loneliness, including the association between hearing loss and loneliness (see Shukla et al., 2020, for a review). On the UCLA-LS, participants respond to statements by indicating how often each statement applies to them. For example, the participant reads the statement “I feel left out,” and responds that they often, sometimes, rarely, or never feel this way. Each response is given a score from 1 (*never*) to 4 (*often*). The scores are then summed. Higher scores indicate greater feelings of loneliness. Scores are treated continuously.

### Audio Recording and SiN Classification

The recorders used were Sony ICD-TX660 digital recorders. The recorders are approximately 2 × 10 × 0.75 cm and weigh 29 g. All features and filters of the recorder were turned off to ensure the most unaltered recording of the acoustic environment possible. Recordings were made in mp3 format (192 kbps; 44.1-kHz sampling rate; stereo signal) so that the recorder memory was sufficient to collect recordings all week (max recording time = 177 hr), and participants did not need to be sent home with multiple devices. The microphones of the recorder are located at the top of the device. Participants were instructed to wear the recorder clipped to the front collar of their shirt or with a lanyard around the neck, with the microphones exposed, as close to the head as possible given their clothing. Participants were given explicit instructions on the wear and use of the recorder. To operate the recorder, participants were instructed to power on the device, press the red record button, ensure the red record light was on, and then switch on a hold button, which locked the buttons on the recorder so that the recorder was not stopped or paused inadvertently. Participants were asked to wear the audio recorder for all waking hours, if possible, and to charge the device at night. Participants were sent home with a carrying case, charger, and an instruction sheet for operating the device. The clock on the recorder was checked prior to sending them out to ensure the correct date and time coding on the recordings.

This study followed the ethics and best practice guidelines for collecting longform audio recordings in daily life proposed by an international working group in Cychosz et al. (2020). In accordance with these guidelines, the following procedures were implemented. Participants were instructed that they could pause or stop the recorder at any time. They were instructed that they should stop the recorder if they needed to, rather than leave the recorder running in a location they were not at. Audio data were extracted and analyzed in an automated way using computer algorithms, rather than transcribed or coded by human listeners. Content of the audio recordings was not analyzed; rather, broad sound classifications (speech, noise, music, silence) were analyzed. Participants were informed of this procedure ahead of the experiment. Recordings were stored in a secured, encrypted drive with access limited only to research staff directly involved with the collection and analysis of the recordings. We made sure that participants understood what would and would not be done with the audio data. They were also informed that they were not obligated to record anything they did not feel comfortable recording, even though human listeners would not code or transcribe those data by hand. Generally, we used recording and analysis procedures similar to those used in studies using the LENA device (e.g., Cristia et al., 2021; Klein et al., 2018; Wu et al., 2019), with the exception that sound classification was performed using an open-source deep neural net rather than the proprietary LENA algorithms.

**Figure 1.**
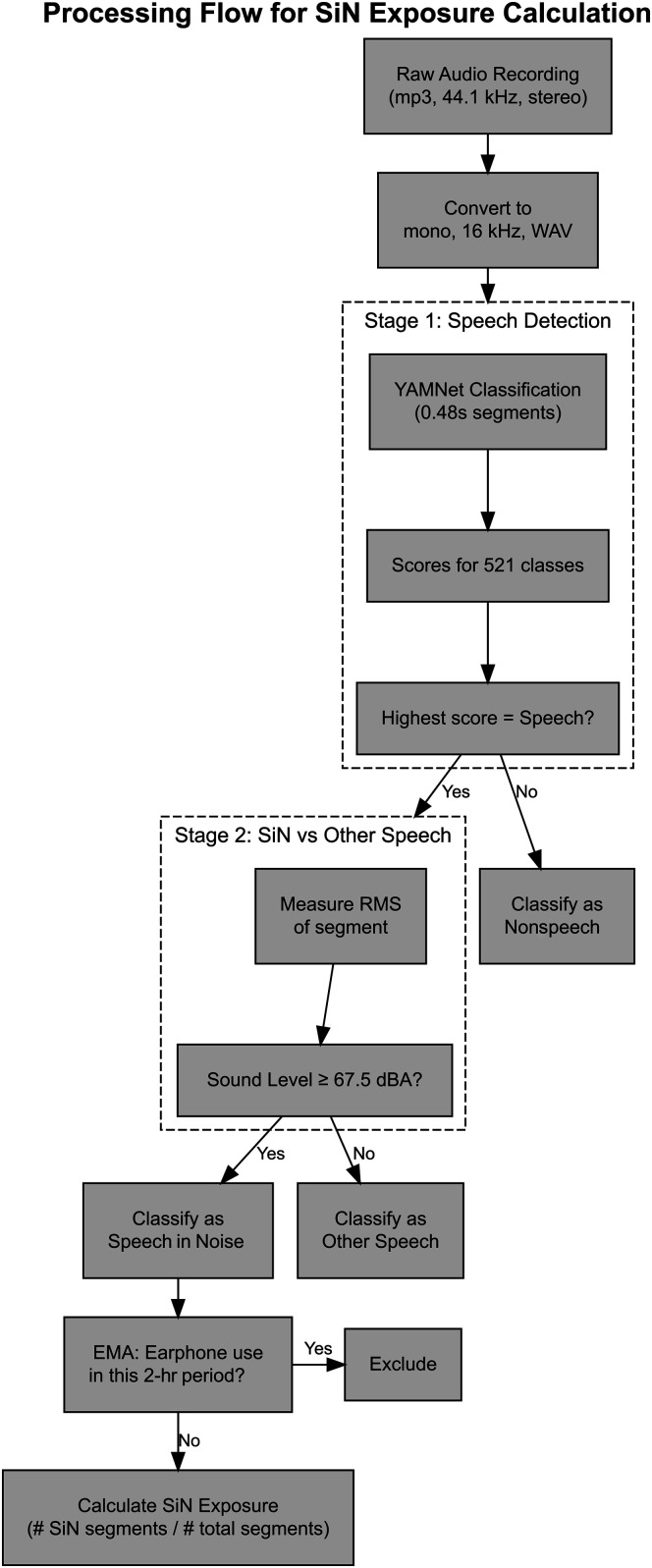
Diagram showing the workflow for processing audio files from the recorder and calculating the proportion of speech-in-noise (SiN) exposure for each participant. YAMNet = Yet Another Mobile Network; RMS = root-mean-square; EMA = Ecological Momentary Assessment.

The audio recordings were analyzed to estimate SiN exposure time, defined as the proportion of time each participant was in a “cocktail party” environment—a multitalker SiN background. We assume for the purposes of this study that these environments are indicative of social situations. SiN exposure was identified through a two-stage process. In the first stage, we identified all sounds that were speech using a deep neural network. In the second stage, we differentiated SiN from other types of speech (conversation in quiet, TV) using a sound pressure level-based criterion. A diagram showing the workflow for processing the audio files and calculating the proportions of SiN time for each participant is shown in Figure 1. Speech sounds were identified using the deep neural network, Yet Another Mobile Network (YAMNet; Google Research, AudioSet Team, 2018; Plakal & Ellis, 2019). YAMNet is a deep learning model developed by Google Research for environmental sound classification. It is a convolutional neural network (CNN) trained on Google's AudioSet, a large-scale data set containing over 2 million labeled audio events across 521 sound categories, including speech, music, environmental noises, and other auditory events (Gemmeke et al., 2017). YAMNet classifies audio by converting the signal into a log-mel spectrogram, passing it through a MobileNet-based CNN architecture, and extracting hierarchical features. The model is pretrained and available for use via TensorFlow Hub. For every 0.48 s of audio, the model returns confidence scores (from 0 to 1) for each of the 521 sound classes in the ontology, where the score corresponds to the confidence that the audio belongs to that sound class. Although performance is category specific, across all 521 classes, YAMNet achieves an overall good performance with a balanced mean average precision of 0.306, a sensitivity index (d-prime) of 2.318, and a label-weighted label-ranking average precision of 0.393 (Plakal, 2019). Details on the training, performance, and implementation of YAMNet are available on the YAMNet GitHub repository (Google Research, AudioSet Team, 2018; Plakal & Ellis, 2019). For this study, YAMNet was run on Python 3. Audio files were processed using the Slurm Workload Manager on a computer cluster, as each participant's data required hundreds of thousands of classifications and the required computational load was high. Analyzing a single participant's data on a regular office computer took days, while running a participant on the computer cluster usually took around 1 hr. YAMNet requires mono WAV files with a 16-kHz sampling rate. Prior to analysis, the 44.1-kHz mp3 stereo recordings were converted to this format. Extensive testing was performed prior to the study to determine whether YAMNet sound class estimations were affected by recording in mp3 and converting to WAV format, and no effects were found—YAMNet classifications were identical whether from recordings originally in WAV format or recordings converted to WAV from mp3. A 0.48-s segment of audio was classified as speech if the highest score returned by YAMNet was for the speech class. These segments were then further processed in a second stage to separate SiN from other instances of speech. YAMNet does have a class for “hubbub, speech noise, speech babble” in its ontology. In practice, YAMNet's confidence scores are evaluated in relation to one another for a given audio class, rather than according to a fixed threshold. During our testing, we observed that YAMNet rarely gave high enough confidence scores to the “hubbub, speech noise, speech babble class” to place the class in the top five classes for testing samples known to be SiN. Rather, YAMNet almost always returned the highest score for the general speech class when the sample was SiN. For the testing samples, the scores for the hubbub, speech noise, speech babble class were low such that they could not be meaningfully used to differentiate SiN from other types of speech. This may be because the speech class had exponentially more annotations in the training set than the hubbub, speech noise, speech babble class (1,010,480 for speech vs. 1,480 for speech noise). We thus implemented a simple sound level–based criterion to distinguish SiN from other speech after YAMNet processing, with the assumption that SiN has typically higher sound levels than speech conversation in quiet or media speech. To develop and test this approach, we purposefully collected 13 hr of recordings in a variety of SiN environments (restaurants, bars, coffee shops, social gatherings) while wearing the recorders in the manner implemented in this study and engaging in conversation. We processed these recordings through YAMNet, which identified 91% of the approximately 97,500 segments in these recordings as being speech. The remaining classifications were random, single incidental noise classifications among long strings of speech classes, such as music, clink, hiss, or machine, indicating brief moments where some other noise besides speech noise became prominent. For each segment classified by YAMNet as speech, we measured the root-mean-square (RMS) of the segment in tandem with the YAMNet classification. We selected a 10th percentile criterion such that 90% of the 0.48-s segments in our test set of SiN recordings had RMS values above this criterion. To determine the corresponding real-world sound level for this RMS criterion, we measured the transfer function of the recorders using pink noise and the protocol described for the LENA device in Wu et al. (2018). The transfer function indicated that the recorders had linear sound level encoding between approximately 35 and 80 dBA, becoming compressive above 80 dBA (and thus very similar to the transfer function of the LENA device). The sound pressure level corresponding to the 10th percentile RMS value for SiN was measured at 67.5 dBA. Thus, SiN was essentially operationally defined for this study as speech sounds above 67.5 dBA. This definition is aligned with many prior studies characterizing real-world sound levels of SiN (e.g., Humes et al., 2018; Pearsons et al., 1977; Rindel, 2010; Sato et al., 2011; Wu et al., 2018). Using this definition, the SiN exposure time for each participant was calculated by dividing the total number of 0.48-s audio segments identified as SiN by the total number of audio segments for that participant.

Classifications of SiN were corrected for earphone use. Not correcting for earphone use in the analyses could overestimate proportions of SiN by including moments in SiN when participants were not actively engaged in listening to the SiN—for example, when participants were in a busy coffee shop but wearing earphones while studying. To account for earphone use in the recording analyses, participants were sent an EMA every 2 hr throughout the week. The EMA asked participants if they had been mostly listening to earphones during the prior 2 hr. When participants indicated they had been mostly listening to earphones, that 2-hr segment was reclassified as speech or music, whichever the participant indicated they had been listening to on their earphones. EMAs were delivered using the ExpiWell app on the participant's own phone. EMAs were delivered approximately 2 hr, with a 15-min random jitter around the 2-hr delivery time (e.g., a survey scheduled for 12:00 p.m. could be delivered anytime between 11:45 a.m. and 12:15 p.m.). Participants were notified of EMA delivery using whatever push notification alert they had set on their phone (e.g., ring, vibrate). Participants were able to choose the delivery time of their first EMA of the day, after which EMAs were delivered every 2 hr for 16 hr until the end of the day. To incentivize compliance, participants were paid $1 for each survey they completed; however, they could not self-initiate surveys. Participants were trained on the EMA at their first appointment and given a practice survey. For more details about the EMA, rationale for measuring earphone use time using EMA rather than other methods, and results about the patterns of earphone use in this sample, see Jorgensen, Reynolds, et al. (2025).

### Statistical Analyses

We used a moderated mediation framework to examine whether the association between time spent in SiN environments and depression operated indirectly through loneliness, and whether this association varied as a function of SPIN ability (Baron & Kenny, 1986). The analytic approach followed established regression-based procedures for moderated mediation (Hayes, 2018; Preacher et al., 2007). We opted for a regression framework rather than structural equation modeling due to the modest sample size, the fact that all variables were observed rather than latent, and the relatively straightforward nature of the proposed relationships. Where appropriate, *p* values are reported, but the primary criteria for significance were confidence intervals (CIs) that excluded zero, as recommended for mediation analyses (Hayes & Scharkow, 2013). First, we tested whether SiN time was associated with loneliness, and whether this association was moderated by SPIN ability. We fit a linear regression model predicting loneliness from SiN time, SPIN ability, and their interaction. A significant interaction would indicate that the association of SiN time with loneliness differed across levels of SPIN ability (first-stage moderation). Second, we examined whether loneliness was associated with depression, controlling for SiN time, by regressing depression on both loneliness and SiN time. This tested the b-path of the mediation model. A significant association would indicate that loneliness is meaningfully related to depression even after accounting for SiN time, supporting its role as a potential mediator in the association between hearing-related behavior and depression. Then, we estimated conditional indirect associations by calculating the product of the conditional a-path (from SiN time to loneliness, moderated by SPIN ability) and the b-path (from loneliness to depression). We used the Johnson–Neyman technique to identify the regions of the moderator (QuickSIN score) where the conditional indirect association was statistically significant. This technique probes the conditional indirect association across the range of the moderator and defines specific regions where the CI excludes zero (Hayes, 2018; Johnson & Fay, 1950). We implemented this procedure using a nonparametric percentile-based bootstrap with 1,000 resamples to estimate CIs for the conditional indirect association at each moderator value (Hayes, 2018). These results allowed us to determine the values of QuickSIN scores at which the indirect association between SiN time and depression through loneliness was statistically significant.

Assumptions of linearity, normality, homoscedasticity, and multicollinearity were checked for all regression analyses. Residual-versus-fitted and scale-location plots supported linearity and homoscedasticity. Q–Q (quantile–quantile) plots showed approximately normal residuals for loneliness (UCLA-LS), while residuals for depression (PHQ-9 scores) showed mild right skew. Data transformations did not substantially improve the distribution or alter the results, so the original scale was retained. Generalized variance inflation factors were computed and were low for all predictors, indicating no concerns with multicollinearity (Fox & Monette, 1992). Because there were no multicollinearity concerns, we chose not to mean-center the predictors to maintain straightforward interpretability. Time spent in SiN was not centered because it is a proportion, and the amount of increase from no time spent in SiN enables useful and meaningful interpretation. Because QuickSIN scores (dB SNR loss) are a deviation from a normative mean, we also left QuickSIN scores on the original scale. All analyses were conducted in R Version 4.5.0 (R Core Team, 2025).

## Results

### Descriptive Statistics

**Figure 2.**
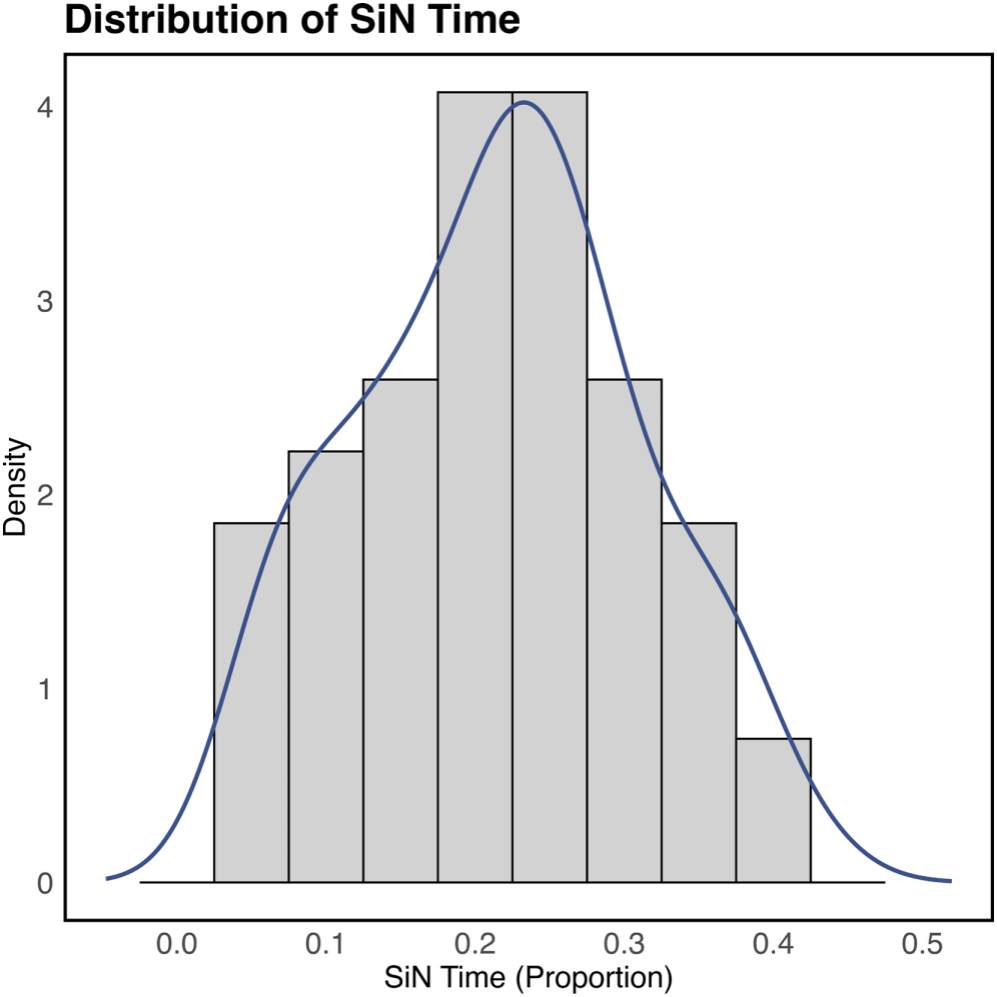
Histogram with probability density function of proportions of time spent in speech in noise (SiN) across participants. Bin width is 0.05 (5%).

Hours of recording data per participant ranged from 32.24 to 129.07 hr, with a mean recording time of 85.25 hr and an *SD* of 21.47 hr. This equates to, on average, 12.18 hr of recordings per day (*SD* = 3.07). Assuming an average of 16 waking hours per day, this equates to a compliance rate of 76%. EMA compliance ranged from 29% to 100%, with an average of 73% (*SD* = 18%). Most participants reported relatively low earphone use. The mean proportion of earphone use was 0.03 (*SD* = 0.05). The minimum proportion was 0 (no earphone use). One participant had considerable earphone use time, at a proportion of 0.27, with the next highest proportion being 0.17. After removing audio classifications during earphone use, SiN exposure was calculated. The mean proportion of time spent in SiN was 0.22 (*SD* = 0.09, min = 0.05, max = 0.42). This equates to, on average, 17.9 total hours over the week (*SD =* 9.27, min = 3.35, max = 36.6) or 2.56 hr per day (*SD* = 1.32, min = 0.48, max = 5.23). Figure 2 presents a histogram and probability density distribution illustrating proportions of time spent in SiN across participants. Mean dB SNR loss on the QuickSIN was 1.68 dB (*SD* = 1.54, min = −1.50, max = 6.00). QuickSIN scores were not correlated with time spent in SiN (*r* = −0.10, *p* = .457), suggesting that participants who spent more time in SiN did not have better SPIN ability.

Responses on the PHQ-9 and UCLA-LS were investigated for potential biases, such as participants responding to the entire questionnaire battery with the same response or moving through the test battery very rapidly (all questionnaires were time-stamped with start and completion times). No evidence of response biases was detected. Reliability was measured for the PHQ-9 and UCLA-LS administered in our sample by computing Cronbach's alpha. The PHQ-9 demonstrated good internal consistency, with a Cronbach's α of .87. The UCLA-LS demonstrated excellent internal consistency, with a Cronbach's α of .93. Examination of item-total correlations suggested that all items contributed positively to the reliability of both scales. The mean score on the PHQ-9 was 4.78 (*SD* = 4.35, min = 0, max = 16) indicating, on average, minimal to mild depression. The mean score on the UCLA-LS was 37.31, indicating, on average, moderate loneliness (*SD* = 11.69, min = 20, max = 71).

### Moderated Mediation Analyses

**Figure 3.**
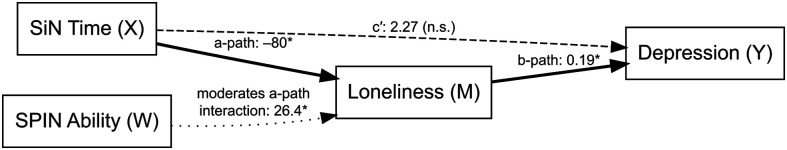
Schematic showing the relationships among variables in the moderated mediation model. The a-path is the direct association between time spent in speech-in-noise (SiN time) and loneliness (UCLA Loneliness Scale score), moderated by speech perception in noise (SPIN) ability (Quick Speech-in-Noise dB SNR loss). The b-path is the direct association between loneliness and depression, controlling for SiN time. The c'-path is the direct association between SiN time and depression (Patient Health Questionnaire-9 score). n.s. = not significant. *Significant at *p* < .05.

A schematic showing the relationships among variables in the moderated mediation model as well as the coefficients for the paths is provided in Figure 3. Asterisks next to coefficients indicate significant relationships. Note that the conditional indirect associations between SiN time and SPIN ability are not shown in the schematic but are provided in the results.

**Figure 4.**
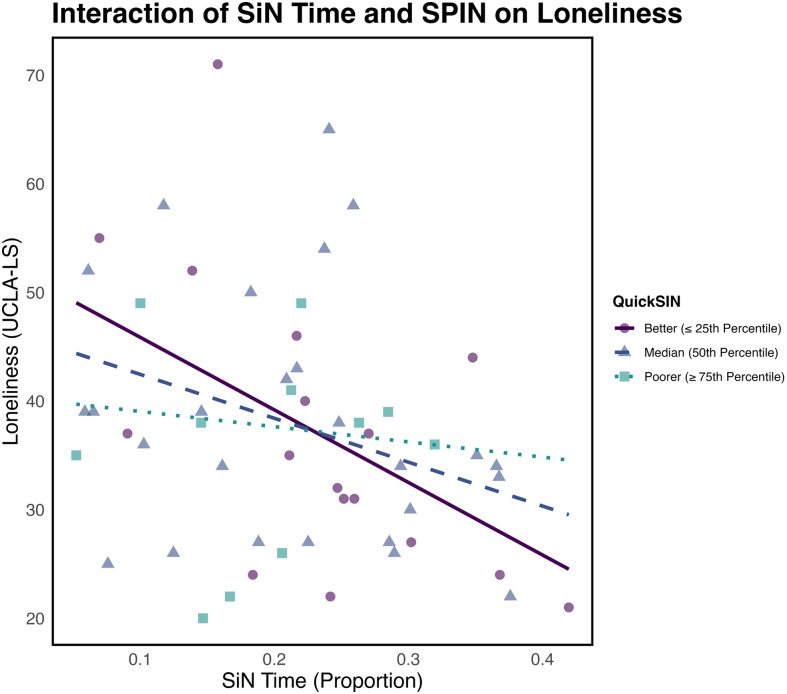
Interaction plot showing the relationship between loneliness (UCLA Loneliness Scale [UCLA-LS] score, *y*-axis) and proportion of time spent in speech in noise (SiN proportion, *x*-axis) for three levels of speech perception in noise (SPIN) ability, as measured on the Quick Speech-in-Noise (QuickSIN) test. Each point represents an individual participant. Lines are modeled regressions. Colors, line types, and individual point shapes indicate QuickSIN score quantile, where lower quantiles indicate better SPIN ability. Lower loneliness is associated with more time spent in SiN, with a larger effect for lower QuickSIN scores.

We first examined whether SiN time was associated with loneliness and whether this association varied as a function of SPIN ability (a-path). The results of the statistical model are shown in Table 1. The overall model was significant, *F*(3, 50) *=* 3.44*, p =* .023. The main effects were significant: Higher percentage of time in SiN and lower QuickSIN scores were significantly associated with lower loneliness scores. More importantly, the interaction between SiN time and SPIN ability was significant, suggesting that the association between SiN time and loneliness depended on SPIN ability. The relationship among SiN time, SPIN ability, and loneliness is shown in Figure 4, where the predicted loneliness as a function of SiN time is shown for three levels of SPIN ability: participants with a low (25th percentile, QuickSIN = 0.5, i.e., better) QuickSIN score, participants with a median QuickSIN score, and participants with a high (75th percentile, QuickSIN = 2.5, i.e., worse) QuickSIN score. The interaction plot shows that a 10% increase in time spent in SiN for participants with a low QuickSIN score was associated with an average 6.68-point decrease in loneliness score. In contrast, a high QuickSIN score was associated with only a 1.4-point decrease in loneliness for the same 10% increase in SiN exposure. Following this, for individuals with very high QuickSIN scores, the relationship reverses, such that increased time in SiN could potentially be associated with higher loneliness scores. The model predicts that this reversal occurs with QuickSIN dB SNR losses greater than 3 dB. The model also suggests that at low SiN exposure times, loneliness scores are lower for individuals with poorer SPIN ability, whereas at high SiN exposure times, loneliness scores are lower for those with better SPIN ability. The model predicts that the crossover occurs at a SiN exposure of 23%. Above SiN times of 23%, individuals with lower QuickSIN scores have lower loneliness than those with higher QuickSIN scores, but below 23%, loneliness is lower for those with higher QuickSIN scores. The model explained 17.1% of the variance in the outcome (*R*^2^ = .17, adjusted *R*^2^ = .12).

**Table 1.** Regression results for the moderated a-path model predicting loneliness (UCLA Loneliness Scale score) from time spent in speech in noise (SiN time) and speech perception in noise (SPIN) ability (Quick Speech-in-Noise dB SNR loss).

Predictor	Estimate	*SE*	*t* value	Pr(>|*t*|)	95% CI
Intercept	55.57	6.03	9.21	< .001	[43.45, 67.69]
SiN Time	−80.00	25.11	−3.19	.002	[−130.42, −29.57]
SPIN Ability	−6.05	2.72	−2.23	.030	[−11.51, −0.59]
SiN Time × SPIN Ability	26.39	11.92	2.21	.031	[2.44, 50.34]

*Note.* Both main effects and their interaction were significant. CI = confidence interval.

**Figure 5.**
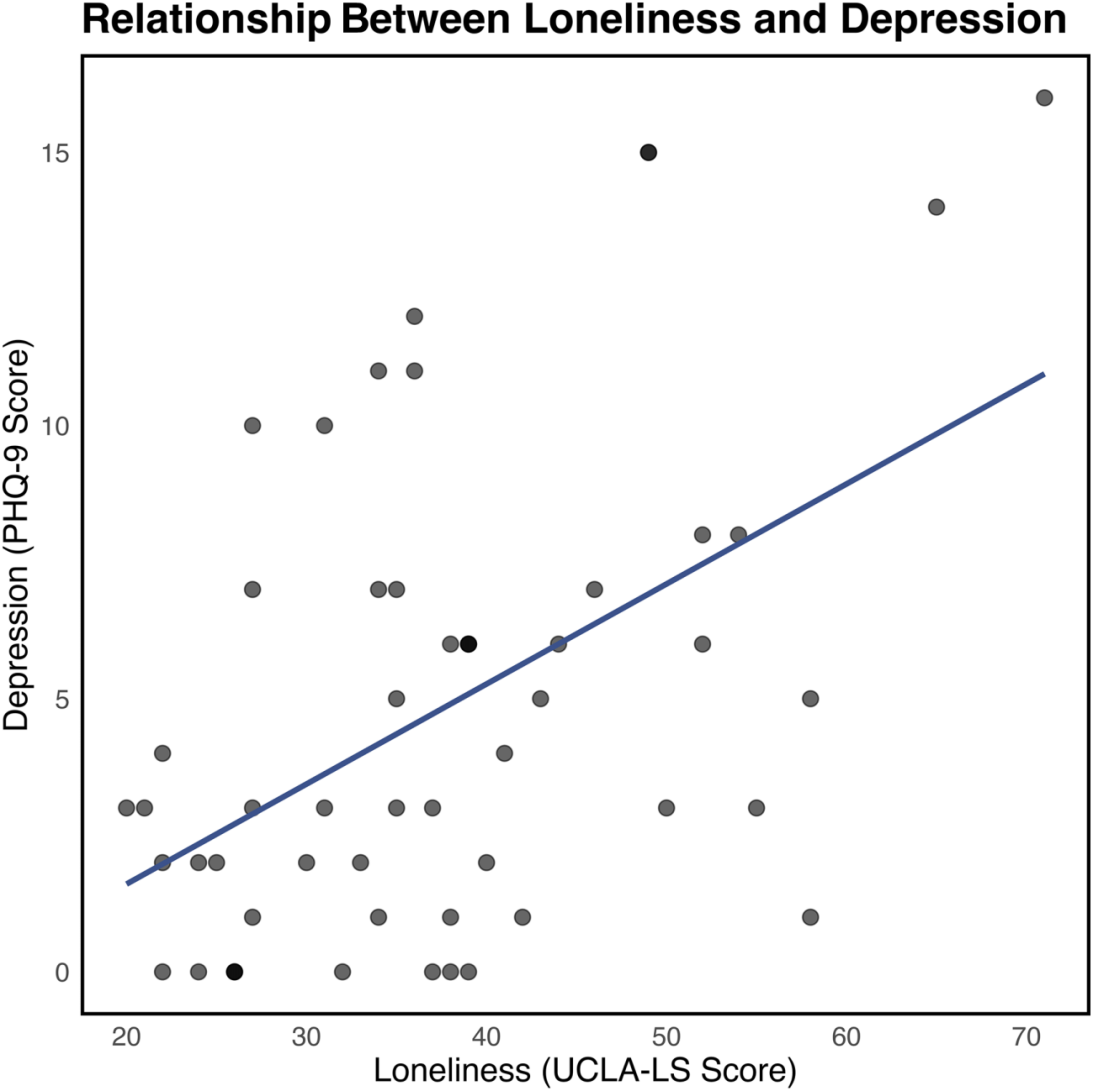
Scatterplot showing the relationship between loneliness (UCLA Loneliness Scale [UCLA-LS] score) and depression (Patient Health Questionnaire-9 [PHQ-9] score). Each point represents an individual participant. The line indicates the modeled regression, demonstrating a significant positive association between loneliness and depression after adjusting for time in speech in noise.

Next, we computed a second linear regression to test whether loneliness was associated with depression after controlling for SiN time (b-path). The association between loneliness and depression is shown in Figure 5. The overall model was significant, *F*(2, 51) = 8.24, *p* < .001. Results showed that higher loneliness was significantly associated with higher depression (*b* = 0.19, *SE =* 0.05, *t =* 3.98*, p* < .001, 95% CI [0.09, 0.28]), while the direct association with SiN exposure was not significant (*p* = .70, 95% CI [−9.63, 14.16]). The model explained 24% of the variance in depression (*R^2^* = .24).

**Figure 6.**
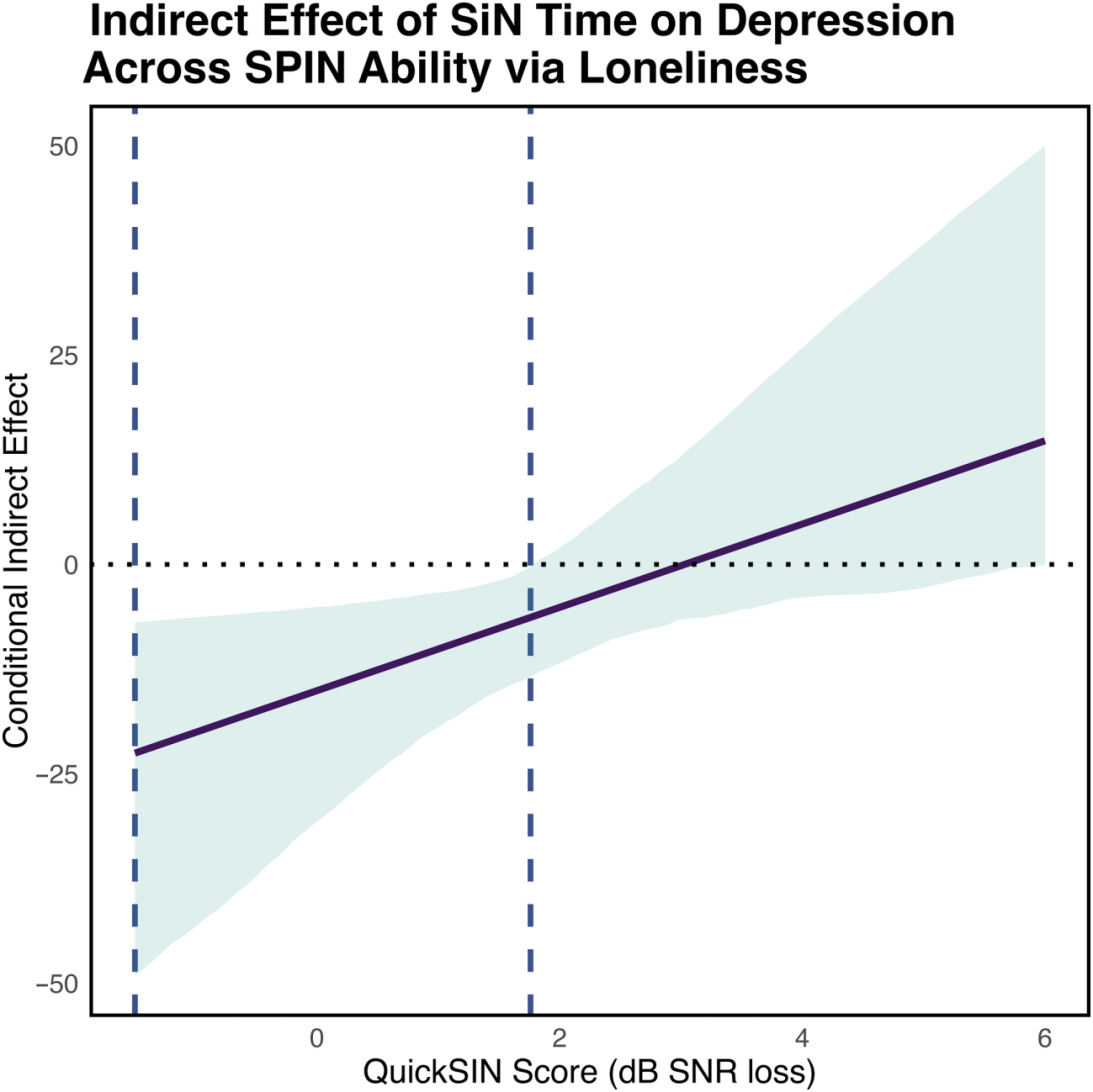
Conditional indirect associations of time spent in speech in noise (SiN) on depression (Patient Health Questionnaire-9 scores) through loneliness (UCLA Loneliness Scale scores), shown across the range of speech perception in noise (SPIN) ability, as measured by Quick Speech-in-Noise (QuickSIN) dB SNR loss scores. Indirect associations were estimated using the Johnson–Neyman technique with bootstrapped 95% confidence intervals (shaded region). The horizontal dotted line at zero indicates the threshold for statistical significance; where the shaded region includes zero, the association is not significant. The vertical dashed lines mark the range of QuickSIN scores for which the indirect association was statistically significant. These results indicate that greater time spent in SiN was indirectly associated with lower depression via reduced loneliness, but only for individuals with approximately average or better SPIN ability.

We then used the Johnson–Neyman technique to examine how the conditional indirect association of SiN time with depression through loneliness varied continuously across the range of SPIN ability. The results of this analysis are shown in Figure 6, where the indirect association of SiN time with depression is plotted as a function of SPIN ability. The vertical dashed lines indicate the interval over which the association is statistically significant (the range of QuickSIN scores for which the bootstrapped 95% CI of the conditional indirect association does not include zero). These conditional indirect associations were calculated as the product of the a-path (SiN time with loneliness, which included an interaction with SPIN ability) and the b-path (loneliness with depression). Because the a-path was moderated, the indirect relationship varies across the range of QuickSIN scores. This analysis revealed that the conditional indirect association was statistically significant for QuickSIN scores between −1.50 and 1.76 dB SNR loss. Outside of this region, the CI included zero, indicating nonsignificance. This finding suggests that the indirect association between more SiN time and lower depression through lower loneliness was present primarily for individuals with approximately average or better SPIN ability. For example, for participants with lower QuickSIN scores (25th percentile), the indirect association was −12.6 (95% CI [−24.4, −4.2]), and for median QuickSIN scores, the association was −7.6 (95% CI [−15.1, −1.4]). For individuals with higher QuickSIN scores (75th percentile), the indirect association was −2.6, which was not statistically significant (95% CI [−8.7, 5.8]). The total association of SiN exposure with depression (c'-path) was not statistically significant (*p* = .471, 95% CI [−17.53, 8.22]), indicating that the association between SiN exposure and depression operated mainly through the indirect pathway via loneliness, with the strength of this pathway moderated by SPIN ability.

Finally, to evaluate whether an alternative direction of influence might explain the observed associations, we tested a reverse mediation model in which depression predicted SiN exposure indirectly through loneliness. The indirect association of depression with SiN time through loneliness was small and marginally significant (indirect association = −0.003, 95% CI [−0.01, 0.00], *p* = .026). The direct association of depression with SiN time, controlling for loneliness, was not statistically significant (direct association = 0.001, 95% CI [−0.01, 0.01], *p* = .718), and the total association was also not statistically significant (total association = −0.002, 95% CI [−0.01, 0.00], *p* = .436).

## Discussion

We examined a moderated mediation model linking time spent in SiN environments, SPIN ability, loneliness, and depression in young adults with audiometrically normal hearing. This study yielded three key findings. First, greater time in SiN environments was associated with lower loneliness, particularly among individuals with better SPIN ability. Second, individuals with poorer SPIN ability showed weaker—or, at very low ability levels, even reversed—associations between SiN exposure and loneliness. A crossover pattern also emerged: Individuals with poor SPIN ability who spent little time in SiN had lower loneliness than individuals with better SPIN ability who also spent little time in SiN. Third, the association between greater SiN exposure and lower depression operated indirectly through loneliness, but only for individuals with approximately average or better SPIN ability. Together, these findings suggest that links between hearing-related behaviors and social–emotional health may depend on the alignment between individuals' hearing abilities and the acoustic demands of the environments that are socially important to them.

These results align with the auditory ecology framework, which emphasizes that the implications of hearing for communication and health outcomes depends on the interactions between the environment and the individual's perceptual capacity (Gatehouse et al., 1999, 2003, 2006). Within this framework, loneliness may arise for some individuals when the demands of the acoustic environments in their social settings do not match their ability to engage successfully in those environments. Our findings are consistent with this framework, suggesting a potential person–environment fit in this context: The association between acoustic environments and social–emotional health may depend on whether an individual's perceptual abilities are sufficient for meaningful participation in the social settings that matter to them. When there is a good fit between a person's SPIN ability and the environments in which their valued relationships occur, the perceived gap between desired and actual social connection may be smaller (Hawkley & Cacioppo, 2010). Individuals who enjoy and value engagement in noisy social settings but have difficulty communicating in those environments may experience a disconnect between desired and realized social engagement, which may increase risk for loneliness. This framework may also help explain mixed findings regarding the benefits of hearing interventions on social–emotional health among older adults. Although speech comprises a large part of older adults' acoustic environments, it is usually in quiet (Humes et al., 2018; Wolters et al., 2016; Wu et al., 2019). Cochlear implants often produce large improvements in audibility and speech understanding in quiet environments. Given that cochlear implant users may experience large perceptual benefits in most of their environments, findings that cochlear implants improve social–emotional health are consistent with this framework (McRackan et al., 2018). In contrast, one of the most common reasons hearing aid users do not wear their hearing aids is that they do not often encounter situations where they need them, which are typically noisy situations (McCormack & Fortnum, 2013). Hearing aids, even with advanced signal enhancement features, provide mixed improvements in SPIN ability (e.g., Wu et al., 2019). If good SPIN ability is important for maintaining the social relationships some individuals seek and, thus, for avoiding a perceived mismatch between desired and actual social connection for those individuals, then the limited effectiveness of hearing aids in precisely these challenging environments may explain why evidence for social–emotional benefits of hearing aid use is inconsistent (Bott & Saunders, 2021; Döge et al., 2025). If true, this could suggest that counseling around hearing aids might benefit from incorporating this perspective, with an emphasis on aligning intervention goals with real-world communication challenges and lifestyles (Timmer et al., 2023). Importantly, we did not investigate the links between SiN exposure, SPIN ability, loneliness, and depression among hearing aid or cochlear implant users in this study, and generalization to older adults or individuals with audiometric hearing loss should be done with caution. Future studies should explicitly investigate the role of hearing loss on hearing-related behaviors and social–emotional health and the interacting effects of hearing technologies.

An incidental but important observation is that young adults in our sample spent roughly twice as much time in SiN as older adults in previous studies (Humes et al., 2018). This is consistent with prior work showing more active auditory lifestyles in younger adults (Jorgensen et al., 2023; Wu & Bentler, 2012). It remains unclear whether reduced SiN exposure with age reflects lifestyle changes, hearing-related behavioral changes, or both. We did not observe a significant association between SPIN ability and SiN time—that is, participants with better SPIN ability did not spend more time in SiN. Thus, we did not find evidence that young adults who have more difficulty communicating in noise withdraw from these environments. In older adults who often experience hearing decline over decades, withdrawal associated with hearing loss may be more evident. Future work could clarify the role of hearing-related behavior changes in mediating age-related declines in social engagement.

Several important limitations should be considered when interpreting the results of this study. First, compliance with both the audio recorder and EMA was imperfect—76% and 73%, respectively. In audiology research, EMA compliance varies widely from approximately 50% to 90% (Holube et al., 2020; Wu et al., 2021). In psychology, where EMA is more common, average compliance is 75% (Jones et al., 2019). To our knowledge, data on compliance with longform, continuous audio recording in adults is limited. In Klein et al. (2018), adults instructed to wear LENA recorders during all waking hours achieved an average of 11 hr of recording per day, slightly less than our observed compliance. We did not ask participants with lower compliance to report reasons for their lower compliance, so it is unclear whether missed recording time was random or systematic. Prior research on EMA compliance in audiology has shown mixed findings on this issue (Schinkel-Bielefeld et al., 2020; Wu et al., 2021). However, reasons for noncompliance likely differ between EMA and passive audio recording. EMA prompts may be missed due to distractions, tasks, or social settings. In contrast, audio recording operates passively, and noncompliance is more likely due to privacy concerns, forgetfulness, or user error in operating the device. It is essential for both ethical and scientific reasons to allow participants to turn off the recorder (Cychosz et al., 2020). Requiring continuous wear to participate might bias the sample toward participants with certain lifestyles, beliefs, or comfort levels with constant monitoring. Allowing opt-outs allows for participant autonomy but comes with the trade-off of imperfect compliance. The statistical models we used assume that data are missing at random, but future studies using this method could specifically investigate this.

Second, although we classified SiN environments using established acoustic criteria and a large sound classification model, we cannot be certain that all SiN environments were social in nature. For example, being in a crowded coffee shop while studying may not involve social engagement. While we corrected for earphone use to help address this issue, future studies should continue to refine measurement approaches to SiN exposure in daily life. Our correction approach for earphone use using EMA was also imperfect. To our knowledge, there is not currently available technology to easily track exact times of earphone use. Emerging technologies like wearable computer glasses, improved sound classification models or other neural network acoustic analyses methods like speaker diarization, or refined EMA approaches that enable better compliance like microEMA could bolster confidence in estimations of SiN exposure. Although we think our classification approach was reasonable given technological constraints, it is difficult to assess its true accuracy, as the ground truth of SiN exposure time is unknown. We used automated classification to maintain participant privacy, get buy-in from our participants, and limit reactivity effects (Hufford et al., 2002). Using automated processes however means that we must base the confidence in our analyses on the reported accuracy of YAMNet and our purposefully collected audio recordings in known SiN environments. We tested other audio classification models (e.g., Listen, Think, and Understand; Gong et al., 2024), but we found that using the two-stage YAMNet and sound level approach was both the simplest and most consistently reliable approach. Continued improvements in audio classification and larger labeled training data sets made specifically for characterizing individuals' acoustic environments will enable future studies that can confirm and refine the findings presented here.

Third, SPIN ability measured in the lab using the QuickSIN may not be indicative of real-world hearing ability or communication success (e.g., Borg et al., 2008; Gatehouse et al., 1999; Jorgensen, Hohmann, & Kayser, 2025; Keidser et al., 2020; Miles et al., 2022; Wu et al., 2019). Communication success in the real world depends on many environmental and perceptual factors not captured by a traditional SPIN assessment. Future studies could expand on the work presented here by more explicitly investigating the interactions between SiN exposure and real-world communication and their potential relationship to social–emotional health outcomes.

Fourth, our study was cross-sectional. The direction of our model (SiN exposure associated directly with loneliness and indirectly associated with depression, moderated by SPIN ability) is grounded in theory, but these data cannot establish the direction of causation. It is possible, for example, that individuals with depression withdraw from social environments, resulting in reduced SiN exposure and increased loneliness. We tested a reverse mediation model to investigate this possibility, and it did not provide strong support for this direction of association. Nonetheless, other directions of associations are possible (e.g., Wagner et al., 2025). Future work using longitudinal designs and randomized controlled trials will be critical to addressing these questions (Döge et al., 2025; Motala et al., 2024).

Lastly, although the moderated mediation associations were statistically significant and bootstrapped CIs were robust, effect sizes were modest, and the CIs were wide in some cases. Our sample size, though reasonable for an intensive ecological study, may have limited power to detect small effects. Using longform audio recordings paired with EMA is powerful in that it provides granular, ecological data, but deploying these methods with the sample sizes often seen in large survey studies would be difficult. The models only accounted for a relatively small portion of the variance in loneliness and depression, suggesting that other important factors were not considered. Loneliness and depression are complex, multifactorial constructs with many contributing factors. We chose to focus specifically on hearing-related behaviors to provide a framework and a method of estimating real-world SiN exposure that can be useful in furthering our understanding of the connections between hearing-related behaviors and social–emotional well-being. We did not include many potentially important factors, such as personality, physical health, social anxiety, cultural heritage, or other demographic factors that may impact auditory lifestyle and social–emotional health (Maes et al., 2019; McClannahan et al., 2025; Mushtaq et al., 2014; Ramírez-Esparza et al., 2024; Schutter et al., 2020). Hearing-related behavior plays only a small part in overall social–emotional health status of a given person. Replication with larger and more diverse samples and the inclusion of additional variables will be important for confirming the effect sizes and the generalizability of these findings.

## Conclusions

This study is an examination of a moderated mediation model linking time spent in SiN environments, SPIN ability, loneliness, and depression. The findings suggest that for some young normal-hearing individuals, spending more time in SiN environments is associated with reduced loneliness, but only for individuals with good (approximately average or better) SPIN ability. For these individuals, greater SiN exposure may be indirectly associated with lower depression via reduced loneliness. Individuals with good SPIN ability but who spend little time in SiN may have higher loneliness than individuals with poor SPIN ability who also spend little time in SiN. This pattern is consistent with a person–environment fit perspective, in which the associations between acoustic environments and social–emotional health depend on how well individuals' hearing abilities align with the types of social settings they encounter. These results highlight the importance of considering both auditory lifestyle and hearing ability when examining the link between hearing and social–emotional well-being. This framework offers a promising approach for future studies investigating how hearing-related behaviors and audiologic interventions may influence social–emotional health.

## Author Contributions

**Erik Jorgensen:** Methodology, Data curation, Formal analysis, Writing – original draft. **Megan Werner:** Methodology, Data curation, Formal analysis. **Lucas Modahl:** Formal analysis. **Sara Misurelli:** Methodology, Writing – review & editing.

## Ethics Statement

This study was approved by the institutional review board of the University of Wisconsin–Madison on September 15, 2023 (2023–0411; Principal Investigator: E. Jorgensen). Written informed consent for inclusion in this research was obtained from all participants.

## Data Availability Statement

The data that support the findings of this study are available on Open Science Framework: https://osf.io/xwajd/.
